# Ocular Sarcoidosis: Contemporary Insights and Future Directions

**DOI:** 10.22336/rjo.2025.77

**Published:** 2025

**Authors:** Călina-Anda Sandu, Vlad Constantin Donica, Cosmin-Victor Ganea, Mădălina-Ioana Bîlha, Anisia-Iuliana Alexa, Camelia Margareta Bogdănici

**Affiliations:** 1 Department of Ophthalmology, “Grigore T. Popa” University of Medicine and Pharmacy, Iași, Romania

**Keywords:** ocular sarcoidosis, dry eye disease, ferning test, biomarkers, FADGs, EDI-OCT, uveitis, OcSar = Ocular sarcoidosis, IWOS = International Workshop on Ocular Sarcoidosis, SUN = Standardization of Uveitis Nomenclature criteria, HRCT = High-resolution computed tomography, 18F-FDG PET/CT = Positron Emission Tomography With 2-Deoxy-2-Fluoro-D-Glucose, sIL-2R = Soluble Interleukin-2 Receptor, ACE = Angiotensin-Converting Enzyme, EDI-OCT = Enhanced Depth Imaging Optical Coherence Tomography, microRNAs = Micro Ribonucleic Acids, FADGs = Fatty Acid Degradation-Related Gene Signatures, EOS = Blau syndrome/early-onset sarcoidosis, NOD2 = Nucleotide-binding oligomerization domain-containing protein 2, DED = Dry Eye Disease, S1T = Schirmer I test, T-BUT = Tear Break-Up Time, MGD = Meibomian Gland Dysfunction, CME = Cystoid Macular Edema, BHL = Bilateral Hilar Lymphadenopathy, ACE = Angiotensin-Converting Enzyme, LCA = Latent Class Analysis, SAU = Sarcoidosis-Associated Uveitis, BAL = Bronchoalveolar Lavage, SS-OCT = Swept-Source OCT, SCT = Subfoveal Choroidal Thickening, HL = Haller’s Layer, FT = Tear Ferning Test, MTX = Methotrexate, AZA = Azathioprine, MMF = Mycophenolate Mofetil, JAK = Janus Kinase Inhibitors, TNF = Tumor Necrosis Factor, PPV = Pars Plana Vitrectomy

## Abstract

**Background:**

Ocular sarcoidosis (OcSar) is a complex manifestation of systemic sarcoidosis that affects more than half of patients. Characterized by non-caseating granulomas, it can involve any ocular structure, from anterior uveitis to optic neuropathy. Its clinical heterogeneity has earned it the nickname “The Great Chameleon”, as diagnosis often requires integration of ophthalmic, systemic, and laboratory findings.

**Methods:**

A targeted literature search was conducted in PubMed for publications from the last five years on OcSar, including clinical studies, systematic reviews, and meta-analyses. Non-English articles, abstracts, and preprints were excluded. Forty-two relevant studies were analyzed to summarize current diagnostic and therapeutic strategies.

**Results:**

Diagnosis is guided by the revised International Workshop on Ocular Sarcoidosis (IWOS-2019) and the Standardization of Uveitis Nomenclature (SUN) criteria. High-resolution computed tomography (HRCT) and positron emission tomography with 2-deoxy-2-fluoro-D-glucose (18F-FDG PET/CT) improve systemic assessment, while soluble interleukin-2 receptor (sIL-2R), angiotensin-converting enzyme (ACE), and lymphopenia support diagnostic accuracy. Enhanced depth imaging optical coherence tomography (EDI-OCT) identifies subclinical choroidal inflammation, and molecular markers such as dysregulated micro ribonucleic acids (microRNAs) and fatty acid-related gene signatures offer potential non-invasive diagnostic tools. The Ferning test also provides insight into tear film alterations associated with ocular sarcoidosis. Treatment progresses from corticosteroids to immunosuppressants (methotrexate, mycophenolate) and biologics (adalimumab, infliximab), with pars plana vitrectomy reserved for refractory or complicated cases.

**Discussion:**

The evolving understanding of ocular sarcoidosis shows that conventional clinical evaluation alone is insufficient to capture the full spectrum of disease activity. Integrating structural imaging with tear-film analysis and molecular profiling not only enhances diagnostic confidence but also reveals previously underrecognized pathways of inflammation. These insights highlight the systemic nature of the disease and underscore the need to reassess monitoring strategies and therapeutic sequencing in light of emerging evidence.

**Conclusion:**

Ocular sarcoidosis remains a diagnostic and therapeutic challenge due to its variability and risk of irreversible vision loss. Combining advanced imaging, molecular biomarkers, and targeted biologic therapies enables precision-based management, improving visual outcomes and patient quality of life.

## Introduction

Sarcoidosis is a systemic inflammatory disease of unknown etiology, histopathologically characterized by the formation of non-caseating granulomas, with a marked predilection for the lungs, lymphatic system, skin, and eyes [**1**]. Ocular sarcoidosis (OcSar) represents the third most frequent manifestation of the disease, affecting approximately 30-60% of patients [**2, 3**]. Although it can occur at any age, OcSar presents a significant diagnostic challenge due to its highly polymorphic clinical spectrum. Sarcoidosis is often described as a “promiscuous disease”, capable of involving virtually any organ system [**4**].

Ocular involvement encompasses a wide array of manifestations, including anterior, intermediate, or posterior uveitis, keratitis, conjunctivitis, adnexal lesions (such as lacrimal gland involvement), and severe complications such as optic neuropathy and neuro-ophthalmic disease [**4, 5**]. This remarkable clinical variability has earned ocular sarcoidosis the epithet “The Great Chameleon”, emphasizing that its diagnosis is frequently one of exclusion [**5**].

Ocular sarcoidosis serves as a sentinel form of the disease, as ocular signs often precede systemic diagnosis in nearly 78.8% of cases, making the ophthalmologist the first point of access for establishing the diagnosis [**4**]. Clinical presentation varies by demographic factors; for instance, Asian (particularly Japanese) and African-American populations show higher rates of ocular involvement [**3**]. The often prolonged delay between the onset of nonspecific symptoms and definitive diagnosis based on clinical criteria and the exclusion of other granulomatous causes can lead to irreversible structural complications and vision loss [**6, 7**].

In light of these challenges, recent research has focused on identifying precision biomarkers and imaging-based screening tools to shorten the diagnostic pathway and facilitate early implementation of steroid-sparing therapeutic strategies [**3, 8**].

The purpose of this review is to summarize recent developments in the literature and provide a structured overview of ocular pathology, encompassing structural, imaging, histological, and biochemical alterations, as well as current therapeutic approaches. The review also aims to serve as a concise guide for diagnostic orientation. A comprehensive PubMed database search was conducted, focusing on studies published within the past five years using “ocular sarcoidosis” as the primary keyword. Relevant prospective and retrospective studies, meta-analyses, and systematic reviews were included, while abstracts, non-English publications, editorial letters, conference posters, and preprints were excluded. A total of 42 articles met the inclusion criteria and were analyzed for this review.

### Heterogeneous Clinical Spectrum and the Need for Proactive Screening

OcSar can affect all segments of the eye, though uveitis, typically granulomatous in nature, remains the predominant manifestation [**4, 9**]. Two main clinical clusters have been described. An acute form, occurring between 20-30 years of age, characterized by active systemic disease and acute uveitis, and a chronic form, generally occurring after the age of 50, most often in Caucasian women, with recurrent uveitis [**10**].

In pediatric populations, Blau syndrome/early-onset sarcoidosis (EOS), caused by NOD2 mutations, leads to severe granulomatous panuveitis with rapid progression and complications [**9**]. In a cohort of newly diagnosed patients with pulmonary sarcoidosis, approximately 27% presented with bilateral uveitis, most commonly panuveitis or posterior uveitis; cutaneous lesions and ocular symptoms were predictive of uveitis, whereas parenchymal lung involvement was inversely associated [**11**].

Typical clinical findings include large “mutton-fat” keratic precipitates (observed in up to 46% of cases), iris nodules (Koeppe or Busacca) [**6, 12**], and trabecular meshwork nodules (in approximately 32.2% of eyes) [**12**]. “Tent-like” anterior synechiae may be seen in up to 35% of patients [**9**].

An often-underestimated component is ocular surface involvement. Systemic sarcoidosis, through granulomatous infiltration of the lacrimal and/or meibomian glands, is strongly associated with the development of dry eye disease (DED). These nonspecific symptoms are frequently managed in isolation, delaying suspicion of a systemic cause. Patients with ocular sarcoidosis demonstrate significantly reduced Schirmer I test values (mean 12.9 ± 10.5 mm vs. 24.3 ± 10.5 mm in controls) and shorter tear break-up times (T-BUT). Studies also show meibomian gland dysfunction (MGD) with higher rates of gland dropout, indicating both aqueous-deficient and evaporative components [**13, 14**].

Cornea: Primary corneal involvement is uncommon. Atypical manifestations include peripheral ulcerative keratitis (PUK), often recurrent and unilateral, which may result in profound thinning (up to 85% stromal depth). Sterile stromal infiltrates or intrastromal granulomas, poorly responsive to empirical antibiotics yet showing dramatic response to corticosteroids, have also been described. Sarcoidosis may predispose to the formation of sterile granulomatous infiltrates in response to foreign bodies, such as corneal sutures [**15**].

Conjunctiva: Conjunctival granulomas may provide a histopathologic confirmation of systemic sarcoidosis [**16**].

Rare Myogenic Ptosis: An uncommon clinical manifestation is ptosis resulting from direct biopsy-proven granulomatous involvement of the levator palpebrae superioris muscle, a myogenic mechanism distinct from more typical causes such as Horner’s syndrome or mechanical compression by an enlarged lacrimal gland [**17**].

### Posterior Segment and Neuro-Ophthalmic Involvement

Posterior segment involvement is most often responsible for severe and irreversible visual sequelae [**7**]. Typical findings include retinal vasculitis (periphlebitis with the classic *candle-wax drippings* appearance) and chorioretinitis. Cystoid Macular Edema (CME): CME secondary to posterior sarcoid uveitis is the leading cause of vision loss and is frequently refractory to conventional corticosteroid therapy [**6**]. Sarcoid Optic Neuropathy: Although rare (1-5% of cases), optic neuropathy may occur as an isolated manifestation, mimicking other causes of optic atrophy, including advanced glaucoma [**5, 18**]. The presentation can be atypical, with bilateral optic neuritis, sudden onset, or primary optic atrophy, underscoring the diagnostic elusiveness of the “sarcoid chameleon” [**5**].

Diagnosis of ocular sarcoidosis relies on a combination of clinical signs, systemic investigations, and biomarkers. The 2019 Revised International Workshop on Ocular Sarcoidosis (IWOS) criteria (**Table 1**) define four levels of diagnostic certainty - definite, presumed, probable, and possible - based on histological confirmation, radiologic evidence of bilateral hilar lymphadenopathy (BHL), and characteristic ocular findings [**9**]. Exclusion of ocular tuberculosis (negative tuberculin skin test or interferon gamma release assay) is mandatory [**10**]. Additionally, the Standardization of Uveitis Nomenclature (SUN) criteria, developed using machine learning algorithms, have been proposed to classify sarcoidosis-associated uveitis with high specificity [**19**].

**Table 1:** 2019 IWOS diagnostic criteria for OcSar

Definite OS	A biopsy-supported diagnosis that is consistent with uveitis.
Presumed OS	A compatible uveitis is present, but a biopsy was not performed. A chest X-ray or CT scan shows BHL, along with at least two positive intraocular findings.
Probable OS	A compatible uveitis is present without a biopsy, and no chest X-ray or CT findings are present. The diagnosis is made if there are at least three intraocular findings and two positive investigational tests.
Possible OS	A compatible uveitis is present with a negative biopsy and no positive chest X-ray or CT findings. This category applies if there are at least four intraocular signs and two positive investigational tests, with or without a negative lung biopsy.
Key systemic investigations	Bilateral hilar lymphadenopathy (BHL) on chest X-ray or CT scan Negative tuberculin or interferon-gamma releasing assays Elevated serum angiotensin-converting enzyme (ACE) Elevated serum lysozyme Abnormal gallium-67 scintigraphy or FDG-PET imaging Lymphopenia Parenchymal lung changes consistent with sarcoidosis

A recent Latent Class Analysis (LCA) applied to IWOS criteria validated a two-class model - Sarcoidosis-Associated Uveitis (SAU) vs. Non-SAU. The presence of vitreous snowballs or “string-of-pearls” opacities demonstrated the highest classification performance (Sensitivity 59.2%, Specificity 95.3%), followed by bilateralism (Sensitivity 90.9%) and BHL (Specificity 94.1%). The combination of the four most discriminative parameters - vitreous snowballs/string-of-pearls, periphlebitis/macroaneurysm, bilateralism, and BHL - achieved a sensitivity of 84.8% and specificity of 95.4% in identifying SAU subtypes [**20**].

### Advanced Imaging Techniques and High-Performance Biomarkers

Systemic Imaging: While BHL detection on chest X-Ray shows limited sensitivity (~50%) [**7**], High-Resolution Computed Tomography (HRCT) provides superior sensitivity (85.7-98.0%) for BHL identification, making it a cornerstone in screening protocols [**9**]. Positron emission tomography with 2-deoxy-2-fluoro-D-glucose (18F-FDG PET/CT) is valuable for assessing systemic inflammatory activity and identifying extrapulmonary biopsy sites [**10, 21**]. Notably, PET/CT can reveal intraocular or subclinical foci of inflammation even in patients with normal thoracic CT findings [**22**].

Serum Biomarkers: The soluble Interleukin-2 receptor (sIL-2R) demonstrated a sensitivity of 98% and a specificity of 94%, outperforming both ACE and chest imaging in some cohorts. ACE remains highly specific (83-99.5%) but with moderate sensitivity (22-73%) [**7, 9**]. Combined sIL-2R and ACE testing enhances diagnostic accuracy [**23**]. Lymphopenia (< 1000 cells/μL) is an essential supportive finding in the revised IWOS criteria, with reported specificity up to 96.7% [**24**].

### Fluid Analysis

Bronchoalveolar lavage (BAL): An elevated T helper cell (CD4/CD8 ratio), > 3.5, is a recognized diagnostic criterion [**10**]; BAL positivity was reported in 67.3% of definite or presumed OS cases [**25**].

Vitreous fluid: The CD4/CD8 ratio in vitreous samples may also provide significant diagnostic value.

Microbial Etiology: *Propionibacterium acnes* has been implicated as a potential microbial trigger in ocular sarcoid granulomas [**26**].

### Choroidal Imaging and Subclinical Screening

Enhanced Depth Imaging Optical Coherence Tomography (EDI-OCT) and Swept-Source OCT (SS-OCT) have revolutionized the ability to visualize the choroidal structure in sarcoidosis.

Subfoveal Choroidal Thickening (SCT): Chronic sarcoid inflammation lasting more than five years has been shown to induce a measurable increase in SCT. Recent studies demonstrate that patients with systemic sarcoidosis, even in the absence of clinically apparent uveitis, exhibit significantly thicker choroids than healthy controls [**27**]. The thickening predominantly involves Haller’s layer (HL), suggesting subclinical choroidal inflammation. These findings position EDI-OCT as a crucial, proactive screening tool capable of identifying patients at risk of ocular involvement before symptomatic disease onset [**28**].

Eyes treated with systemic corticosteroids exhibit significantly reduced choroidal thickness compared to those under observation, supporting the hypothesis of inflammation-driven structural remodeling [**28**].

Typical Imaging Signs: Additional useful imaging features include macular edema, choroidal nodules, and vitreous “snowballs/string-of-pearls” opacities, the latter of which are among the best-performing parameters for classifying sarcoid uveitis [**20**].

### Advanced Molecular Biomarkers

Definitive diagnosis of sarcoidosis traditionally relies on histopathological confirmation (e.g., lymph node, skin, or conjunctival biopsy). Yet, current research is increasingly focused on non-invasive diagnostic biomarkers, circulating or local molecular signatures that can support early diagnosis and disease monitoring.

Fatty Acid Degradation-Related Gene Signatures (FADGs): A significant recent advance has been the identification of distinct gene-expression signatures associated with FADG pathways that are dysregulated in ocular sarcoidosis. Using advanced regression algorithms, a diagnostic model based on these hub genes distinguished OcSar eyes from healthy controls with high diagnostic accuracy, suggesting a previously unrecognized metabolic component in disease pathogenesis. Transcriptomic profiling identified genes such as ADH1B and ECI1 as highly discriminative (AUC = 1.000) in distinguishing OcSar from controls, providing novel insight into metabolic dysregulation in sarcoid pathophysiology [**4, 29**].

miRNA Analysis: Comprehensive analysis of vitreous miRNA expression has revealed a specific dysregulation pattern in ocular sarcoidosis. These altered miRNAs are involved in key signaling cascades, particularly the TGF-β pathway, which modulates both inflammation and granulomatous processes [**30**]. Such profiles may serve as novel diagnostic and prognostic biomarkers, offering potential for targeted molecular diagnostics and therapeutic monitoring.

Lacrimal Gland Dysfunction: Sarcoidosis is frequently associated with DED. Studies demonstrate significantly lower Schirmer I Test (S1T) values in OcSar patients (12.9 ± 10.5 mm/5 min) compared with both Vogt-Koyanagi-Harada-related uveitis and healthy controls, while tear meniscus height remains normal. This suggests a dysfunction localized to the main lacrimal gland, part of the neural reflex arc, whereas accessory gland secretion remains intact [**13**].

Tear Ferning Test (FT): The non-invasive FT, which evaluates tear-film crystallization patterns, has demonstrated distinctive alterations in OcSar. Microscopic assessment reveals a linear crystallization pattern with a significantly increased branching score, reflecting altered tear biochemical composition under systemic inflammation. The Masmali score showed 70% sensitivity and 75% specificity for sarcoid-related DED, suggesting a unique tear-film signature in this condition [**31**].

### Management of Refractory Disease and Vitreoretinal Surgery

First-line treatment of active OcSar remains topical, periocular, or systemic corticosteroids [**4**]. However, the chronic and recurrent nature of the disease necessitates long-term steroid-sparing strategies to prevent corticosteroid-related complications such as cataract and glaucoma [**19**].

For chronic or refractory disease, immunosuppressive agents are introduced to reduce steroid dependence. Methotrexate (MTX) is the preferred first-line immunomodulator, ideally administered subcutaneously. Other agents include azathioprine (AZA) and mycophenolate mofetil (MMF), while leflunomide (LFN) serves as an alternative in MTX intolerance. Hydroxychloroquine (HCQ) may be considered, particularly in anterior or intermediate uveitis, though evidence remains limited [**32**].

Biologic Therapy is used in refractory, vision-threatening, or corticosteroid-dependent uveitis. Anti-TNF-α agents (Adalimumab, Infliximab) are the cornerstone of modern management for posterior and panuveitis that are unresponsive to standard immunosuppression (e.g., MTX, MMF). Infliximab has shown superior efficacy in controlling chronic inflammation, achieving steroid-sparing effects, and improving long-term visual prognosis [**33**].

Janus Kinase Inhibitors: JAK inhibitors, such as Filgotinib (in clinical trials) and Tofacitinib, represent emerging options for non-infectious uveitis and pulmonary sarcoidosis. Filgotinib demonstrated efficacy in non-infectious uveitis, whereas Tofacitinib has achieved complete resolution of ocular and mediastinal sarcoidosis in isolated refractory cases [**4**].

Pars Plana Vitrectomy (PPV): Indicated for refractory CME, dense vitreous opacities, or epiretinal membrane formation. PPV provides dual benefits: removal of the inflammatory vitreous milieu (diagnostic and therapeutic vitrectomy) and management of structural complications, including peeling of epiretinal membranes. Early surgical intervention is a critical determinant for optimizing visual outcomes in severe and recalcitrant cases [**3, 6**].

Ocular sarcoidosis remains a major clinical challenge due to its heterogeneous presentation, diagnostic complexity, and high potential for irreversible visual loss. Despite advances in IWOS/SUN diagnostic criteria [**9,19**], a significant proportion of cases lack histologic confirmation, underscoring the need to integrate imaging and molecular diagnostics into routine clinical practice.

EDI-OCT technology enables the early detection of subclinical choroidal inflammation, reshaping diagnostic paradigms and facilitating timely intervention [**27, 28**]. In parallel, miRNA profiling and metabolic gene-signature analysis (FADGs) [**29, 30**] offer promising avenues for identifying precision biomarkers that distinguish active from latent disease and predict therapeutic response.

Lacrimal gland involvement and tear-film compositional changes [**13, 17**] reaffirm the systemic nature of ocular sarcoidosis and justify vigilant monitoring of dry-eye symptoms, which may precede manifest uveitis.

Therapeutically, OcSar management should follow a stepwise approach, from corticosteroids and conventional immunosuppressants (MTX, MMF) to biologic anti-TNF-α agents (adalimumab, infliximab) [**32,33**], which yield significant benefit in refractory forms. In advanced cases, PPV can restore ocular stability and improve visual function.

Looking ahead, targeted therapies and JAK signaling pathways may redefine the control of chronic inflammation [**4, 30**].

## Conclusions

Overall, the integration of non-invasive tests (FT, T-BUT, S1T), modern imaging modalities (EDI-OCT), molecular biomarker discovery, and personalized biologic therapy provides a solid framework for improving visual prognosis. OcSar thus stands as a paradigmatic inflammatory disease in which the fusion of clinical, imaging, and molecular insights transforms management from empirical to precision medicine, ultimately enhancing patient outcomes.

## References

[1] Sève P, Pacheco Y, Durupt F, Jamilloux Y, Gerfaud-Valentin M, Isaac S, Boussel L, Calender A, Androdias G, Valeyre D, El Jammal T (2021). Sarcoidosis: A Clinical Overview from Symptoms to Diagnosis. Cells.

[2] Aoki T, Yokoi N, Nagata K, Deguchi H, Sekiyama Y, Sotozono C (2022). Investigation of the relationship between ocular sarcoidosis and dry eye. Sci Rep.

[3] Takase H (2022). Characteristics and management of ocular sarcoidosis. Immunol Med.

[4] Pasadhika S, Rosenbaum JT (2015). Ocular Sarcoidosis. Clin Chest Med.

[5] Jha S, Pendyala SK, Tiwari M (2024). Neuro-sarcoidosis with isolated optic neuropathy: unmasking the chameleon. The Egyptian Journal of Internal Medicine.

[6] Suzuki K, Ishihara M, Namba K, Ohno S, Goto H, Takase H, Kawano S, Shibuya E, Hase K, Iwata D, Mizuuchi K, Kitaichi N, Mizuki N, Ishida S (2022). Clinical features of ocular sarcoidosis: severe, refractory, and prolonged inflammation. Jpn J Ophthalmol.

[7] O’Keefe GAD, Rao NA (2022). Progress in the diagnosis of ocular sarcoidosis. Indian J Ophthalmol.

[8] Leinonen S (2024). Ocular sarcoidosis, to screen or not to screen?. Front Med (Lausanne).

[9] Bazewicz M, Heissigerova J, Pavesio C, Willermain F, Skrzypecki J (2023). Ocular sarcoidosis in adults and children: update on clinical manifestation and diagnosis. J Ophthalmic Inflamm Infect.

[10] Giorgiutti S, Jacquot R, El Jammal T, Bert A, Jamilloux Y, Kodjikian L, Sève P (2023). Sarcoidosis-Related Uveitis: A Review. J Clin Med.

[11] Lee JH, Han YE, Yang J, Kim HC, Lee J (2023). Clinical manifestations and associated factors of uveitis in patients with pulmonary sarcoidosis: a case control study. Sci Rep.

[12] Nagahori K, Keino H, Nakayama M, Watanabe T, Ando Y, Hayashi I, Abe S, Okada AA (2022). Clinical features and visual outcomes of ocular sarcoidosis at a tertiary referral center in Tokyo. Graefes Arch Clin Exp Ophthalmol.

[13] Kiyat P, Palamar M, Gerceker Turk B (2021). Dry eye and Meibomian gland dysfunction evaluation in sarcoidosis patients. Eur J Ophthalmol.

[14] Eroğul Ö, Balcı A, Gobeka HH, Efe N, Akdoğan M, Oral AY, Doğan M, Özdemir Ç, Kaşıkçı M, Saraçlı S (2023). Conjunctival Impression Cytology and Tear Film Changes in Sarcoidosis: A Subjective and Objective Diagnosis Study. Turk J Ophthalmol.

[15] Córdoba A, Mejía LF, González N, Gil JC (2023). Unusual Corneal Sarcoidosis Manifestations. Ocul Immunol Inflamm.

[16] Zina S, Bouraoui S, Khouaja S, Messaoud M, Maalej A, Letaief H (2022). Conjunctival granulomas leading to the diagnosis of systemic sarcoidosis. J Fr Ophtalmol.

[17] Firn KA, Hawy E, Van Putten DJ (2025). Biopsy-proven sarcoidosis lesion of the levator palpebrae superioris causing myogenic blepharoptosis. Am J Ophthalmol Case Rep.

[18] Jung EH, Kim W, Yoon RG, Kim KE (2023). Coexistence of open-angle glaucoma and sarcoidosis-associated optic neuropathy. BMC Ophthalmol.

[19] Standardization of Uveitis Nomenclature (SUN) Working Group (2021). Development of Classification Criteria for the Uveitides. Am J Ophthalmol.

[20] Xiong F, Acharya N, Rao N, Mochizuki M, Lietman TM, Gonzales JA (2024). Ocular Signs and Testing Most Compatible with Sarcoidosis-Associated Uveitis: A Latent Class Analysis. Ophthalmol Sci.

[21] Zhang Y, Du SS, Zhao MM, Li QH, Zhou Y, Song JC, Chen T, Shi JY, Jie B, Li W, Shen L, Zhang F, Su YL, Hu Y, Lower EE, Baughman RP, Li H (2022). Chest high-resolution computed tomography can make higher accurate stages for thoracic sarcoidosis than X-ray. BMC Pulm Med.

[22] Chauvelot P, Skanjeti A, Jamilloux Y, de Parisot A, Broussolle C, Denis P, Ramackers JM, Giammarile F, Kodjikian L, Seve P (2019). 18F-fluorodeoxyglucose positron emission tomography is useful for the diagnosis of intraocular sarcoidosis in patients with a normal CT scan. Br J Ophthalmol.

[23] Ishihara M, Meguro A, Ishido M, Takeuchi M, Shibuya E, Mizuki N (2020). Usefulness of Combined Measurement of Serum Soluble IL-2R and Angiotensin-Converting Enzyme in the Detection of Uveitis Associated with Japanese Sarcoidosis. Clin Ophthalmol.

[24] Cotte P, Pradat P, Kodjikian L, Jamilloux Y, Seve P (2021). Diagnostic value of lymphopaenia and elevated serum ACE in patients with uveitis. Br J Ophthalmol.

[25] Allegri P, Olivari S, Rissotto F, Rissotto R (2022). Sarcoid Uveitis: An Intriguing Challenger. Medicina (Kaunas).

[26] Okazaki F, Wakiguchi H, Korenaga Y, Nakamura T, Yasudo H, Uchi S, Yanai R, Asano N, Hoshii Y, Tanabe T, Izawa K, Honda Y, Nishikomori R, Uchida K, Eishi Y, Ohga S, Hasegawa S (2021). A novel mutation in early-onset sarcoidosis/Blau syndrome: an association with Propionibacterium acnes. Pediatr Rheumatol Online J.

[27] Byg KE, Ellingsen T, Wied J, Peiris M, Lowater SJ, Sejbaek T, Grauslund J (2025). Increased retinal thickness in sarcoidosis patients with ocular system involvement visualized with optical coherence tomography: a cross-sectional study. Rheumatol Int.

[28] Han YE, Jo J, Kim HC, Lee J (2024). Choroidal manifestations of non-ocular sarcoidosis: an enhanced depth imaging OCT study. BMC Ophthalmol.

[29] Wu Z, Gao Y, Tan K, Yao X, Peng Q, Li W (2025). Fatty acid degradation-related gene signatures as biomarkers for ocular sarcoidosis. Sci Rep.

[30] Asakage M, Usui Y, Komatsu H, Maruyama K, Nezu N, Shimizu H, Tsubota K, Yamakawa N, Umezu T, Takanashi M, Kuroda M, Goto H (2025). Comprehensive microRNA analyses using vitreous humor of ocular sarcoidosis. Graefes Arch Clin Exp Ophthalmol.

[31] Sandu CA, Ganea CV, Donica VC, Alexa AI, Sandu IA, Bilha MI, Bogdanici CM (2025). The Clinical Value of the Ferning Test in Monitoring Dry Eye Syndrome in Patients with Sarcoidosis. Life.

[32] Rao A, Hwang J, Wen J, Edminster S, Sibug-Saber M, Rao N, Toy BC (2024). Clinico-pathologic correlation in ocular sarcoidosis. Am J Ophthalmol Case Rep.

[33] Maalouf G, Andrillon A, Leclercq M, Sève P, Bielefeld P, Gueudry J, Sené T, Titah C, Moulinet T, Rouvière B, Sène D, Desbois AC, Domont F, Touhami S, Thibault T, Chamieh CE, Cacoub P, Kodjikian L, Biard L, Bodaghi B, Saadoun D (2022). Lower Relapses Rate With Infliximab Versus Adalimumab in Sight-Threatening Uveitis: A Multicenter Study of 330 Patients. Am J Ophthalmol.

